# Real-world effectiveness of vonoprazan-based regimen versus rabeprazole-based dual therapy for *Helicobacter pylori* eradication in a high-resistance, multi-ethnic region: a multicenter study in Guizhou, China

**DOI:** 10.3389/fmed.2026.1801129

**Published:** 2026-04-10

**Authors:** Yurong Huang, Juan Zheng, Zhengyi Yang, Daili Li, Hua Wu, Wenzhong Chen, Wenjuan Wang, Min Gan, Changmei Shi, Dongmei Xie, Canyu Zhan, Gengqing Song, Jie Yang

**Affiliations:** 1 Department of Gastroenterology, The Affiliated Hospital of Guizhou Medical University, Guiyang, Guizhou, China; 2 Department of Gastroenterology, Liupanshui People's Hospital, Liupanshui, Guizhou, China; 3 Department of Gastroenterology, Tongren People’s Hospital, Tongren, Guizhou, China; 4 Department of Gastroenterology, Xingyi People’s Hospital, Xingyi, Guizhou, China; 5 Department of Gastroenterology, Anshun People’s Hospital, Anshun, Guizhou, China; 6 Department of Gastroenterology and Hepatology, MetroHealth Medical Center, Case Western Reserve University, Cleveland, OH, United States

**Keywords:** dual therapy, efficacy, *Helicobacter pylori*, rabeprazole, vonoprazan

## Abstract

**Introduction:**

While vonoprazan-amoxicillin dual therapy has achieved > 90% eradication rates for *Helicobacter pylori (H. pylori)* in clinical trials, its effectiveness in “real-world” regions with high primary antibiotic resistance and ethnic diversity remains under-verified. This multicenter study evaluated whether simplified vonoprazan-based regimens can overcome these regional barriers compared to standard rabeprazole-based dual therapy.

**Methods:**

This retrospective analysis (April 2022–November 2023) included 598 *H. pylori*-positive outpatients from 10 centers across 8 prefectures of Guizhou. Patients received one of four 14-day therapies: Group A (vonoprazan 20 mg b.i.d. + amoxicillin 1 g t.i.d.), Group B (vonoprazan 20 mg q.d. + amoxicillin 1 g t.i.d.), Group C (vonoprazan 20 mg q.d. + amoxicillin 1 g b.i.d. + furazolidone 100 mg b.i.d. + colloidal bismuth pectin 100 mg t.i.d.), Group D (rabeprazole 20 mg b.i.d. + amoxicillin 1 g t.i.d.). The primary endpoint was the eradication rate, confirmed by 13C/14C urea breath tests at least 4 weeks post-treatment.

**Results:**

After excluding patients lost to follow-up and those with poor compliance, the eradication rates were 87.2% (Group A), 81.4% (Group B), 82.1% (Group C), and 71.5% (Group D). Group A achieved a significantly higher eradication rate than Group D (*p* < 0.001), while no other significant between-group differences were observed. However, none of the regimens reached the 90% “optimal” threshold. Notably, the once-daily (q.d.) vonoprazan regimens (Groups B and C) failed to reach the 85% clinical acceptability threshold in this specific geographic cohort. No significant association was observed between treatment success and age, sex, or ethnicity.

**Conclusion:**

In the high-resistance environment of Guizhou, vonoprazan-based regimens are significantly more effective than traditional rabeprazole-based dual therapy, yet they face unique regional challenges. The failure of q.d. vonoprazan dosing and the sub-90% success rate of b.i.d. dosing suggest that in regions with extreme resistance and high ethnic diversity, standard global protocols may require further optimization.

## Introduction

*Helicobacter pylori (H. pylori)* colonization of the gastric epithelium is a primary driver of gastrointestinal disease and the most significant modifiable risk factor for gastric cancer (1). Despite a global decline in *H. pylori* prevalence from 52.6% in the 1980s to approximately 43.9% in the last decade, the absolute burden of infection remains substantial, affecting billions worldwide (2). Effective eradication is critical for reducing both the morbidity of gastric diseases and the global mortality associated with gastric cancer (1). Over the past decades, standard treatments have evolved from dual therapy to triple therapy and, more recently, to bismuth-based quadruple therapies (3). However, global eradication efforts are losing efficacy due to rising antibiotic resistance, complex regimens, and poor patient compliance (4).

In response to these challenges, high-dose dual therapy comprising amoxicillin and a potent acid suppressant has emerged as a viable alternative (5). Amoxicillin maintains low resistance rates in China (0–5%) (6), is rapidly absorbed and cleared within 6–8 h (7), and exhibits stable antibacterial activity at gastric pH > 6 (8). High-dose dual therapy has demonstrated eradication rates comparable to or superior to those of other standard treatments (including bismuth quadruple therapy, BQT), with fewer side effects and lower costs (9, 10). Consequently, recent Chinese consensus guidelines recommend it as a first-line or salvage option for *H. pylori* eradication (11).

Potassium-competitive acid blockers (P-CABs), such as vonoprazan, offer distinct advantages over traditional proton pump inhibitors (PPIs), including more rapid, potent, and sustained acid suppression that is independent of CYP2C19 genotypes (12, 13). In Japan, a 7-day regimen of vonoprazan (20 mg b.i.d.) combined with amoxicillin (1.5 g/day) achieved nearly 90% eradication rates (14). In contrast, initial Chinese trials utilizing similar regimens showed mixed results. For instance, a randomized controlled trial (RCT) assessing vonoprazan (20 mg b.i.d.) plus amoxicillin (1 g b.i.d. or t.i.d.) for 7 or 10 days reported Intention-to-treat (ITT) eradication rates of 66.7–89.2%, failing to meet the 90% efficacy threshold (15). To address this shortfall, subsequent single-center studies extended the duration of high-dose dual therapy (vonoprazan 20 mg b.i.d. + amoxicillin 1 g t.i.d.) to 14 days, thereby surpassing the 90% eradication benchmark (16, 17).

While vonoprazan-amoxicillin dual therapy is currently revolutionizing the global landscape of *H. pylori* eradication, its clinical efficacy remains subject to regional heterogeneities in strain virulence and antibiotic susceptibility (18). Guizhou Province is characterized by a unique multi-ethnic demographic and distinct *H. pylori* virulence profiles. Unlike regions where amoxicillin resistance remains negligible, Guizhou has reported a rising trend in resistance, reaching 13.27%, which may undermine the synergistic effect of P-CAB-mediated acid suppression (19). Furthermore, the local prevalence of high-virulence East Asian *cagA*-ABD genotypes (74.1%) presents a significant biological barrier, as these strains are associated with more aggressive mucosal colonization and heightened inflammatory responses (20, 21). While Vonoprazan’s efficacy is established in controlled trials, its performance in ‘real-world’ high-resistance pockets with diverse ethnic compositions—such as Guizhou Province—remains unverified. Guizhou’s unique socio-demographic profile and high rates of primary antibiotic resistance necessitate localized validation of simplified dual therapies against traditional quadruple regimens.

To date, no large-scale, multicenter studies have validated vonoprazan-based regimens in this specific regional context. Therefore, we conducted a multicenter retrospective study in Guizhou Province to evaluate the real-world effectiveness of three vonoprazan-based regimens (high-dose dual, single-dose dual, and quadruple therapy) compared to a rabeprazole-based dual regimen. We sought to determine if these regimens could achieve the preferred ≥90% eradication threshold and to identify risk factors for treatment failure in the Guizhou population.

## Methods

### Study design and ethics

This was a multicenter, retrospective, real-world study conducted between April 2022 and November 2023. Data were aggregated from the gastroenterology departments of 10 hospitals, including 8 tertiary-grade A hospitals and 2 secondary hospitals, across 8 prefectures in Guizhou Province, China. The study protocol adhered to the Declaration of Helsinki and was approved by the Ethics Committees of all participating hospitals (Approval No: LPSSYY-2023-103).

### Participants

We screened outpatients presenting to gastroenterology departments at 10 hospitals. Inclusion criteria were as follows: (1) age ≥ 18 years; (2) confirmed *H. pylori* infection by ^13^C or ^14^C urea breath test (UBT); and (3) documented completion of one of the four designated anti-*H. pylori regimens*. In addition, we excluded participants with the following conditions: (1) use of P-CAB, PPIs, bismuth compounds, or antibiotics within the month following *H. pylori* eradication treatment; (2) known allergy to any study medication; (3) follow-up UBT performed less than 4 weeks post-treatment; (4) history of partial or total gastrectomy; and (5) previous *H. pylori* eradication treatment.

### Treatment regimens

Eligible patients received one of the following four 14-day regimens based on physician discretion and clinical practice at the time:

Group A (High-dose VA Dual): vonoprazan 20 mg b.i.d. + amoxicillin 1 g t.i.d.
Group B (Standard-dose VA Dual): vonoprazan 20 mg q.d. + amoxicillin 1 g t.i.d.
Group C (VA Quadruple): vonoprazan 20 mg q.d. + amoxicillin 1 g b.i.d. + furazolidone 100 mg b.i.d. + colloidal bismuth pectin 100 mg t.i.d.
Group D (PPI-Dual Control): rabeprazole 20 mg b.i.d. + amoxicillin 1 g t.i.d.

The traditional PPI-based standard (Group D) used a b.i.d. schedule for the acid inhibitor, while the standard-dose Vonoprazan group (Group B) used a q.d. schedule. Colloidal bismuth pectin was administered at a dose of 100 mg three times daily (t.i.d.), according to the manufacturer’s instructions and established clinical protocols at our hospital network. This dosage is consistent with common clinical practice in China, where specific dosing standards for bismuth pectin are determined by local formulation specifications. All medications used were standardized across centers: Vonoprazan (Tianjin Wutian Pharmaceutical), Furazolidone (Yunpeng Pharmaceutical), Rabeprazole (Jincheng Haisi Pharmaceutical), Colloidal Bismuth Pectin (Yunpeng Pharmaceutical/Guilin Huaxin Pharmaceutical), and Amoxicillin (Sichuan Yike Pharmaceutical/Shandong Lukang Pharmaceutical).

While Group A and Group D allow a direct comparison of acid-suppression efficacy (vonoprazan vs. rabeprazole), Groups B and C represent pragmatic evaluations of distinct clinical regimens used in regional practice. We acknowledge that the simultaneous variation of antibiotic frequency and the addition of bismuth/furazolidone in Group C precludes the isolation of individual drug effects.

### Data collection

Comprehensive clinical data were retrospectively extracted from the standardized multicenter electronic medical record (EMR) systems. To ensure the integrity of the dataset, two researchers independently extracted data, with a third senior investigator cross-checking for consistency and resolving any discrepancies by trained researchers at each participating center. These variables included demographics (age, gender, and ethnicity), clinical symptoms, esophagogastroduodenoscopy (EGD) findings, treatment regimen, treatment course, *H. pylori* eradication results, therapeutic adherence, and adverse events (AEs).

All patients were managed according to a standardized clinical protocol. Treatment compliance was assessed at the follow-up visit (4 weeks post-therapy) using pharmacy records and pill counts. Adherence was calculated as the percentage of prescribed doses consumed (number of pills actually taken/total number of pills prescribed). Good compliance was defined as taking at least 80% of the prescribed medication. The primary outcome was the *H. pylori* eradication rate, assessed via UBT at least 4 weeks post-treatment. We included patients who completed ≥ 80% of the medication and the follow-up test. Patients who were lost to follow-up, failed to undergo retesting, or demonstrated poor compliance were excluded from the final analysis. A negative UBT result indicated successful eradication.

### Statistical analysis

Data compilation and summary were performed using Excel 2010. Statistical analyses were conducted using R software (version 4.3.2). Continuous variables were reported as mean ± standard deviation (SD) or median (quartiles), depending on their distribution. Categorical variables were presented as frequencies and percentages. Differences in eradication rates between groups were compared using Chi-square tests or Fisher’s exact tests as appropriate. To control for inflation of Type I error rates in multiple comparisons, *p*-values for *post hoc* pairwise comparisons were adjusted using the Bonferroni correction, with a significance level set at *p* < 0.0083 (0.05/6). Both univariate and multivariate logistic regressions were employed to identify factors associated with eradication failure (age, gender, and ethnicity). A *p*-value <0.05 was considered statistically significant.

## Results

### Study population and baseline characteristics

**Figure 1:**
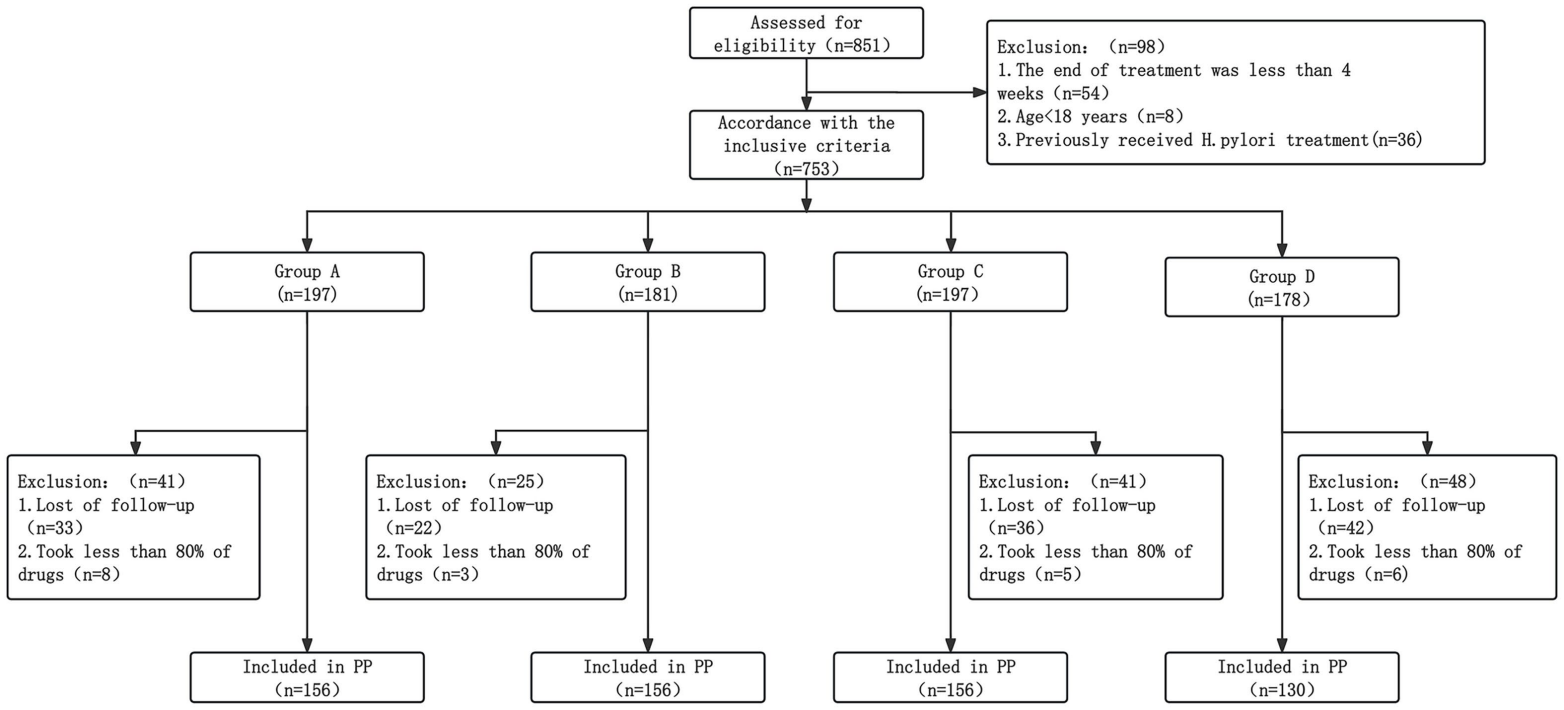
Flowchart of patient recruitment and study disposition. The diagram illustrates the selection process, including total patients screened, exclusion criteria, and the final distribution of participants into groups A, B, C, and D.

A total of 851 patients were initially screened for inclusion. After excluding pediatric patients (*n* = 8), those with inadequate treatment duration (*n* = 54), and those with a history of *H. pylori* eradication therapy (*n* = 36), 753 participants were followed. After further excluding individuals who were lost to follow-up or demonstrated poor medication adherence (<80%), 598 patients were included in the final analysis (Figure 1).

The final cohort was distributed across four treatment arms: Group A (*n* = 156), Group B (*n* = 156), Group C (*n* = 156), and Group D (*n* = 130). The median age across all study groups was approximately 48 years. Baseline characteristics, including age, gender, ethnic distribution, and primary clinical symptoms, were well-balanced across all groups, with no significant differences observed after Bonferroni correction (*p* > 0.0083). However, statistically significant differences were observed in EGD findings (*p* < 0.0083), likely reflecting the multicenter recruitment across diverse regions of Guizhou (Table 1).

**Table 1:** Baseline demographic and clinical characteristics of the study population.

Characteristics	Group A (*n* = 156)	Group B (*n* = 156)	Group C (*n* = 156)	Group D (*n* = 130)	*F/χ^2^*	*P*-value
Age (median, IQR)	49.0(38.0,57.0)	47 (34.8,55.0)	48 (36.8,57.0)	48.5(33.3,57.0)	0.275	0.844
Age cohorts (*n*/%)						
<60 years	125 (80.1)	133 (85.3)	132 (84.6)	108 (83.1)	1.754	0.625
≥60 years	31 (19.9)	23 (14.7)	24 (15.4)	22 (16.9)		
Gender (*n*/%)						
Female	84 (53.9)	96 (61.5)	88 (56.4)	80 (61.5)	2.731	0.435
Male	72 (46.2)	60 (38.5)	68 (43.6)	50 (38.5)		
Ethnicity (*n*/%)						
Han	97 (62.2)	100 (64.1)	97 (62.2)	69 (53.1)	1.833	0.176
Non-Han	59 (37.8)	56 (35.9)	59 (37.8)	61 (46.9)		
Symptom (*n*/%)						
Abdominal pain and /or bloating	91 (58.3)	104 (66.7)	112 (68.0)	88 (67.7)	24.218	0.148
Asymptomatic	36 (23.1)	31 (19.9)	26 (16.7)	24 (18.5)		
Others^b^	29 (18.6)	21 (13.5)	18 (11.5)	8 (6.2)		
Endoscopic findings, *n* (%)						
Gastritis	93 (59.6)	109 (69.9)	102 (65.4)	76 (58.5)	28.351	0.005
Peptic Ulcer^c^	5 (3.2)	18 (11.5)	14 (9.0)	10 (7.6)		
No EGD performed	58 (37.2)	29 (18.6)	40 (25.6)	44 (33.9)		

EGD, esophagogastroduodenoscopy. ^a^Includes Yi, Hui, Chuanqing, Mongol, Maonan, Dong, Tujia, Bai, Shui, She, Gaoshan, and Man. ^b^Includes nausea/vomiting, decreased appetite, hiccups, and acid regurgitation. ^c^Includes gastric ulcer, duodenal ulcer, and combined gastric/duodenal ulcers.

### *Helicobacter pylori* eradication efficacy

The eradication rates for the four regimens are detailed in Table 2. The eradication rates were 87.2% (136/156) in Group A, 81.4% (127/156) in Group B, 82.1% (128/156) in Group C, and 71.5% (93/130) in Group D. To account for multiple comparisons against the control, a Bonferroni-corrected significance threshold of *p* < 0.0167 (0.05/3) was applied for post-hoc pairwise testing. Under this rigorous criterion, Group A (high-dose vonoprazan dual therapy) demonstrated a significantly superior eradication rate compared to the Group D control (rabeprazole-based dual therapy) (87.2% vs. 71.5%; *p* < 0.001). In contrast, while Groups B and C showed higher absolute eradication rates than the control, these differences did not reach statistical significance after adjustment (*p* > 0.0167). Notably, despite the superior performance of the vonoprazan-based regimens, none of the study groups achieved the optimal 90% eradication threshold.

**Table 2:** *Helicobacter pylori* eradication rates across treatment groups.

Group	Success (n)	Total (N)	Eradication rate (%)	*χ* ^2^	*P*-value (vs. Control)
Group A	136	156	87.2%	10.871	<0.001*
Group B	127	156	81.4%	3.893	0.048
Group C	128	156	82.1%	4.462	0.035
Group D (Control)	93	130	71.5%	Ref	Ref

*P*-values reflect comparisons against the control group (Group D). Significance is defined as *P* < 0.0167 after Bonferroni correction for three comparisons. *Indicates statistical significance.

### Risk factors for eradication failure

Both univariate and multivariate logistic regression analyses were utilized to evaluate potential risk factors for treatment failure, excluding individuals with unknown ethnicity. The results showed that age, gender, and ethnicity were not significantly associated with eradication outcomes across the study groups in the final model (*p* > 0.05).

## Discussion

In China, the increasing prevalence of antibiotic-resistant *H. pylori* strains has challenged the efficacy of traditional bismuth-containing quadruple therapy (BQT). Recent Chinese studies have demonstrated that Vonoprazan-based regimens—particularly Vonoprazan-amoxicillin dual therapy—achieve eradication rates exceeding 90%, even in regions with high clarithromycin resistance (22–24). To the best of our knowledge, this is the first multicenter study to evaluate the clinical effectiveness of vonoprazan-based regimens for *H. pylori* eradication in the multi-ethnic Guizhou Province of China. Our findings demonstrate that a 14-day high-dose dual regimen (vonoprazan 20 mg b.i.d. + amoxicillin 1.0 g t.i.d.) significantly outperforms traditional PPI-based dual therapy. However, the failure of any regimen to reach the 90% eradication benchmark highlights a critical “regional gap” in current therapeutic strategies. Our findings extend the utility of vonoprazan-amoxicillin dual therapy beyond homogenous clinical trial populations. In a region like Guizhou, with high primary antibiotic resistance and ethnic diversity, the transition to a dual-therapy regimen that bypasses these resistance pathways is not merely a confirmation of theory but a critical shift in regional public health strategy.

### The rationale for vonoprazan-amoxicillin dual therapy

The recommended first-line BQT has shown declining eradication rates, driven by increasing antibiotic resistance, the need for complex regimens, notable adverse effects, and poor adherence (25, 26). As a result, high-dose PPI plus amoxicillin dual therapy has emerged as a promising alternative approach due to potent acid suppression, low amoxicillin resistance, and robust efficacy (27). Because amoxicillin stability and bacterial replication are optimal at a gastric pH > 6 (8), the use of vonoprazan offers a distinct advantage over traditional PPIs by providing rapid, sustained, and genotype-independent acid suppression (9, 10). This therapy creates a more stable environment for amoxicillin efficacy.

### Comparative efficacy and regional gap

The superiority of vonoprazan over rabeprazole in our study aligns with the pharmacological advantages of P-CABs, which provide more rapid, potent, and sustained acid suppression than traditional PPIs (27). However, a significant “Guizhou Gap” was observed: the eradication rates for the vonoprazan-amoxicillin dual therapy regimens (87.2% in Group A and 81.4% in Group B) were lower than the >90% rates reported in Japan (28, 29) and other regions of China (16, 17, 30).

This discrepancy highlights the impact of regional heterogeneity. While some literature suggests that amoxicillin resistance in Guizhou may be as high as 13.27% (19)—significantly above the national average of <5%—our study did not perform patient-level culture-based antibiotic susceptibility testing (AST). Therefore, while regional antibiotic resistance is a plausible hypothesis for the sub-90% eradication rate, it cannot be invoked as a definitive causal driver without prospective molecular or phenotypic validation. If amoxicillin resistance is indeed the primary barrier in this high-resistance, multi-ethnic population, the most rational clinical solution is a shift toward susceptibility-guided therapy (SGT) (31). By identifying the resistant fraction through pre-treatment testing, clinicians can move beyond empiric “regimen optimization” and instead provide targeted antibiotic substitution.

However, we recognize that in the resource-limited settings of many Guizhou prefectures, the infrastructure for universal culture-based AST remains a challenge. In such pragmatic contexts, our results serve as a critical evidence-based benchmark: they demonstrate that, while vonoprazan-based dual therapy is superior to PPIs, once-daily (q.d.) dosing is insufficient. Until SGT becomes widely accessible across the province, optimizing empiric regimens—specifically by mandating twice-daily (b.i.d.) dosing of vonoprazan—remains the most effective immediate strategy to bridge the therapeutic gap in this unique regional cohort.

### Dose–response relationship

We observed a dose–response relationship with vonoprazan. Increasing the vonoprazan dose (Group A b.i.d. vs. Group B q.d.) showed a trend toward higher eradication rates (87.2% vs. 81.4%), though this was not statistically significant. Group A was more effective than the double-dose rabeprazole dual-therapy control, whereas Group B lost significance. Conversely, the rabeprazole group performed poorly, reinforcing that standard PPIs are insufficient for dual therapy, likely due to inadequate 24-h pH control compared to P-CABs (32). This suggests that in Southwest China, the double-dose vonoprazan frequency is likely necessary to maintain the pH threshold required for amoxicillin to function against more resilient strains.

### Quadruple therapy performance

Given the low resistance of *H. pylori* to furazolidone and amoxicillin in China (6, 30), this study also evaluated a quadruple-therapy regimen (Group C) comprising a single dose of vonoprazan, bismuth, and furazolidone. However, this quadruple therapy did not demonstrate superior efficacy compared to other therapies, with poor performance. A key finding of our study is the ‘functional equivalence’ of Groups B and C (81.4% vs. 82.1%). Although the experimental design does not allow us to determine the specific contribution of furazolidone or bismuth in Group C, the lack of superior efficacy suggests that the added complexity of a quadruple regimen does not offset the benefit of once-daily vonoprazan in this high-resistance environment. This further reinforces the importance of dosing frequency (b.i.d.) over the mere addition of more antimicrobial agents.

This contrasts with studies in Zhejiang Province, where single-dose vonoprazan quadruple therapies achieved > 90% eradication (30), underscoring regional differences in resistance or drug metabolism. Ideally, the addition of furazolidone should mitigate amoxicillin resistance. However, the use of single-dose vonoprazan (20 mg q.d.) in this quadruple regimen may have provided insufficient acid suppression to optimize antibiotic efficacy (33). The disparity suggests that in regions such as Zhejiang, lower resistance levels permit reduced dosing, whereas in Guizhou, the high-resistance environment makes even quadruple therapy challenging. Future studies in Guizhou should investigate whether incorporating a double dose of vonoprazan into the quadruple regimen yields better outcomes.

### Ethnicity

Interestingly, while we hypothesized that Guizhou’s multi-ethnic composition might influence treatment outcomes, logistic regression showed that ethnicity was not an independent predictor of treatment failure. This may be attributed to vonoprazan’s metabolic pathway, which bypasses CYP2C19 polymorphisms that often cause ethnic-based variability in PPI efficacy. This finding further suggests that the efficacy of vonoprazan is independent of genetic polymorphisms in drug metabolism (e.g., *CYP2C19*) (12, 13). Still, vonoprazan-based regimens in Guizhou yielded lower eradication rates than those reported elsewhere in China. Future investigations should consider ethnic factors and differences in drug metabolism between Guizhou and other provinces to clarify these observations.

### Limitations

Several limitations should be acknowledged. First, the study design limits the mechanistic interpretability of certain comparisons. Aside from the direct comparison between Groups A and D, other cohorts involved multiple simultaneous changes in variables (e.g., dosing frequency and antibiotic combinations), which preclude the isolation of the individual contributions of furazolidone or bismuth. Future research should utilize factorial design trials or head-to-head comparisons of single-variable changes (e.g., vonoprazan b.i.d. dual therapy vs. vonoprazan b.i.d. quadruple therapy) to precisely quantify the additive value of bismuth or secondary antibiotics in high-resistance regions. Second, the retrospective design introduces potential selection and recall biases, which may affect the precision of our reporting. Future large-scale prospective RCTs are needed in the Guizhou region to validate these real-world findings and provide higher-level evidence for regional guideline development.

Third, the absence of patient-level AST precludes a definitive causal link between treatment failures and regional resistance. While the “Guizhou Gap” (sub-90% eradication) may be driven by elevated amoxicillin resistance, this remains speculative without phenotypic or genotypic data. Future efforts in Guizhou should prioritize SGT via culture-based AST or molecular screening as the most rational path to achieving optimal eradication thresholds in this multi-ethnic population. Finally, while our study confirms the efficacy of 14-day regimens, it was not designed to determine the minimum effective dosing frequency (e.g., 7-day vs. 14-day) or the impact of varying vonoprazan doses. Future head-to-head trials focusing on dose optimization in high-resistance regions are warranted.

## Conclusion

In the high-resistance landscape of Guizhou Province, a 14-day high-dose vonoprazan-amoxicillin dual therapy (b.i.d.) offers a statistically significant advantage over traditional rabeprazole-based regimens, demonstrating robust safety and high adherence across diverse age and ethnic cohorts. However, our findings reveal a critical “therapeutic gap” between localized clinical outcomes and international standards; even under per-protocol analysis, no regimen achieved the 90% optimal eradication threshold. This indicates that the unique antibiotic resistance profile and socio-demographic factors in Guizhou render a “one-size-fits-all” global approach insufficient.

Most notably, this study establishes that once-daily (q.d.) vonoprazan dosing is clinically inadequate for this region, failing to meet the 85% acceptability threshold. While optimizing dosing frequency to twice-daily (b.i.d.) is a necessary, immediate step for empiric treatment, the persistent sub-90% success rate suggests that future clinical strategies must prioritize transitioning to SGT. Implementing pre-treatment culture-based AST or molecular resistance screening is the most rational path to overcoming regional resistance barriers. Until such infrastructure is widely accessible, high-dose vonoprazan dual therapy remains the preferred empiric benchmark for refining *H. pylori* protocols in high-resistance, multi-ethnic frontier regions.

## Data Availability

The original contributions presented in the study are included in the article/supplementary material, further inquiries can be directed to the corresponding authors.
